# Supplementary material for: Identification of Chalcone Isomerase Family Genes and Roles of CnCHI4 in Flavonoid Metabolism in Camellia nitidissima
Source: Biomolecules. 2022 Dec 26;13(1):41. doi: 10.3390/biom13010041 (PMC9855375; doi:10.3390/biom13010041)
Supplement: Supplementary file 1 [file biomolecules-13-00041-s001.zip › supplement table S1-S7.pdf]

Supplementary Table S1. Sequences of primers used for gene cloning.

Target	Forward primer (5' to 3')	Reverse primer (5' to 3')
CnCHI4 (to pMD19-T)	GTGCTGAGAGAGAAAGAGAGCA	ACTCCACAAGACACATGTAGCT
CnMYB7 (to pMD19-T)	GTTAATCTTCATCTATAGTGTCCTCCA	CCCAAATTCCTCCAACTAATGCA
	ATGGGACGGTCTCCTTGCTG	TCATTTCATCTCCAAGCTTCTGTAAT

Supplementary Table S2. Sequences of primers used for overexpression and subcellular localization.

Target	Forward primer (5' to 3')	Reverse primer (5' to 3')
CnCHI4 (to Pcambia1302)	CATG CCATGG ATGTCTCCATCACAGTCACCGTC	GG ACTAGT TTCAGCAGCAGCAGCTGTCT
CnMYB7 (to pCAMBIA1302)	CATG CCATGGGACGGTCTCCTTGCTG	GA AGATCT GC TTTCATCTCCAAGCTTCTGTAATCC

Supplementary Table S3. Sequences of primers used for the genomic DNA PCR of *Nicotiana tabacum*.

Target	Forward primer (5' to 3')	Reverse primer (5' to 3')
HYG	ATGAAAAAGCCTGAACTCACCG	CTATTTCTTTGCCCTCGGACG

Supplementary Table S4. Sequences of primers used for the qRT-PCR.

Target	Forward primer (5' to 3')	Reverse primer (5' to 3')
NtActing	TCCTGATGGGCAAGTGATTAC	TTGTATGTGGTCTCGTGGATTC
CnGAPDH	GGGAATCCTTGGTTACACTGAG	ACCCCATTCGTTGTCATACC
CnCHI4	GTCCGTCACCCAAGTCCAGAT	TGCCCGGAGGTTTCACA

Supplementary Table S5. Sequences of primers used for promoter cloning.

Order	Order	Forward primer (5' to 3')	Reverse primer (5' to 3')
1	(1)	GGTTCCAAGGCTTTGCTTATGGT	CAGTCTTGCCCTTCCACTTAACG
	(2)	CCGGTTTTTATGATTTGGTTCTG	GCGCTATCTTCGAGGTACACTCC
	(3)	GGAAAAAGATAGCAATGGTGGAG	AAACGTGGCTCTCGATCTGGACT
2	(1)	GGTTCCAAGGCTTTGCTTATGGT	GAGTTGGGTTTGAAGTGATGTGTT
	(2)	CCGGTTTTTATGATTTGGTTCTG	TAATAGCATCCGTGAAGATTGAAA
3	(1)	TCCGGTTTTTATGATTTGGTTCTG	CAGTCTTGCCCTTCCACTTAACG
	(2)	TCCGGTTTTTATGATTTGGTTCTG	GCGCTATCTTCGAGGTACACTCC
	(3)	TCCGGTTTTTATGATTTGGTTCTG	AAACGTGGCTCTCGATCTGGACT

Supplementary Table S6. Sequences of primers used for dual-luciferase assays.

Target	Forward primer (5' to 3')	Reverse primer (5' to 3')
CHI4pro (to pGreen II0800-LUC)	GG GGTACC TCCGGTTTTTATGATTTGGTTCTG	CG GGATCC TGCTCTCTCTTTCTCTCTCAGCAC
CnMYB7 (to pGreenII 62sk)	ACGC GTCGAC ATGGGACGGTCTCCTTGCT	GG GGTACC TCATTTCATCTCCAAGCTTCTGTAA

Supplementary Table S7. Sequences of primers used for Y1H assays.

Target	Forward primer (5' to 3')	Reverse primer (5' to 3')
CHI4pro (to pHIS2)	GGAATTC AATTGTTCCATTGTTCACCAATAAT	TCC CCCGGGAGTTAGAAAGCGGCACAAGAGGG
CnMYB7 (to pGADT7)	GGAATTC CATATG GGACGGTCTCCTTGCT	CG GGATCC TCATTTCATCTCCAAGCTTCTGTAA
